# Early onset cancer trends and the persistently higher burden of cancer in young women

**DOI:** 10.1093/oncolo/oyaf084

**Published:** 2025-05-16

**Authors:** Rebecca D Kehm, Mary Beth Terry

**Affiliations:** Department of Epidemiology, Mailman School of Public Health, Columbia University, New York, NY, 10032, USA; Herbert Irving Comprehensive Cancer Center, Columbia University Irving Medical Center, Columbia University, New York, NY, 10032, USA; Department of Epidemiology, Mailman School of Public Health, Columbia University, New York, NY, 10032, USA; Herbert Irving Comprehensive Cancer Center, Columbia University Irving Medical Center, Columbia University, New York, NY, 10032, USA; Silent Spring Institute, Newton, MA, 02460, USA

Although there are many favorable cancer trends including decreases in overall incidence and mortality, there remains substantial heterogeneity in these trends by race, ethnicity, and age. In particular, there are well-documented and major health disparities, particularly in higher cancer mortality, seen for Black, Hispanic, and Indigenous Americans compared to White Americans.^1^ More recently, there has been growing recognition of the increasing incidence rates of cancers in adults under the age of 50 years (hereafter referred to as early onset cancer).^2^ What is less discussed are the sex differences in the early onset cancer burden. The International Agency for Research on Cancer estimated that currently 2/3 of all cancers diagnosed under age 50 years in the world are diagnosed in women.^3^ However, since this estimate is based on cross-sectional data, it is unclear how the female-to-male incidence ratio has changed over time due to varying incidence rates for many cancer types. In this commentary, we present an overview of the current evidence on early-onset cancer incidence trends. Using the latest data from the Surveillance Epidemiology and End Results (SEER) and United States Cancer Statistics (USCS) public use databases, we analyze sex-specific trends in early-onset cancer, overall, and for the top 10 cancer sites in the United States. We conclude by discussing the potential public health and clinical implications of these trends.

## State of the evidence on early onset cancer incidence trends

In the United States, the overall incidence of early-onset cancers in total has been increasing ever since the SEER registries started counting cancer rates in the 1970s. The overall increase, however, has not been seen for all cancers. For example, during the 1990s there was a rise and fall of HIV-associated cancers.^2^ Some cancers, however, like breast cancer, have been increasing even before the 1970s. For example, using data from the Connecticut cancer registry which started recording cancer incidence in the 1930s, we found that breast cancer incidence rates before age 40 years have been increasing since the 1930s, long before the baby boom.^4^ In contrast, we found that early-onset colorectal cancer has been increasing only in more recent decades in the United States.^5^ These patterns emphasize the importance of conducting disaggregated analyses of cancer trends by cancer type to better understand and address the specific factors contributing to the incidence of each cancer.

The rise in early-onset cancer incidence is not limited to the United States; it is a global phenomenon. The global burden of early onset cancer incidence surpassed 3.26 million in 2019, a 79.1% increase from the incidence in 1990.^6^ It is projected that early onset cancer incidence will continue to increase globally over time, with projections estimating a 31% increase from 2020 to 2030. In addition to the global rise in early-onset cancer incidence, early-onset cancer deaths increased by 28% between 1990 and 2019 and are projected to increase by another 21% from 2020 to 2030.^6^ Several early-onset cancers, including breast, colorectal, kidney, and thyroid cancers have increased in many other parts of the world.^7^ For example, we evaluated trends in early onset breast cancer incidence using global data and found that incidence increased from 1990 to 2017 in 147 of the 185 (79%) countries that we analyzed.^8^ We also found that early onset breast cancer mortality increased in most regions of the world from 1990 to 2017, ranging from a 6% increase in South Asia to a 21% increase in East Asia and the Pacific.^8^

The increases in early onset cancers, an age group in which cancers are rarer, stand in direct contrast to the more stable trends in adults aged 55 to <70 years, and the decreasing trends in cancer incidence in males ≥70 years.^2^ Thus, the overall average can obscure the major heterogeneity in cancer incidence trends across age groups, especially given that more cancer is diagnosed in men than women ≥55 years.^2^ Yet, women <55 years are much more likely to be diagnosed with cancer than men and the rates of change over time have been faster in women than in men.^2^ Therefore, it is important to analyze early-onset cancer incidence trends separately from older age groups when assessing differences in the cancer burden between sexes.

## Evaluation of sex-specific trends in early onset cancer incidence in the United States

Using 45 years of US data, we examined whether this sex difference in early-onset cancers is new and/or has been seen alongside the increase in early-onset cancers witnessed in both men and women. We also examine the individual types of cancer that are contributing to the sex difference in the overall burden of early-onset cancer. We used data from the SEER 8 database, which covers approximately 8% of the US population,^9^ to calculate the proportion of early-onset cancer cases (diagnosed 20-49 years) that were in females for each year from 1975 to 2021. We then used joinpoint regression to examine more recent trends (2001-2019) in age-adjusted (standardized to the 2000 US standard population) early-onset cancer incidence rates by sex and cancer type using data from the USCS, which represents approximately 99% of the US population.^10^ We considered the top ten most common cancer types in adults under 50 years, classified using SEER’s Site recode ICD_0-3/WHO_2008 variable. This included 6 cancers in both males and females (colorectal, thyroid, kidney and renal pelvis, melanoma of the skin, non-Hodgkin lymphoma, and leukemia), 3 cancers in females only (corpus uterine, including not otherwise specified, cervix uteri, and breast), and one cancer in males only (testis). Joinpoint regression models allowed for up to 3 joinpoints and a minimum of 4 observations between adjacent inflection points.^11^ We calculated the average annual percent change (AAPC) using a weighted average of the slope coefficients of the underlying joinpoint regression line with the weights equal to the length of each segment over the interval.^11^ To determine whether the AAPC was statistically different from zero (*P* < .05), a two-sided *t*-test was used for 0 joinpoints and a two-sided z-test was used for 1 or more joinpoints.^11^ Rates were considered to increase or decrease if *P* < .05; otherwise, rates were considered stable between 2001 and 2019. Trends were calculated using Joinpoint software version 5.1.0.0 (released April 2024) from the National Cancer Institute.^12^ We were approved for data usage from a US Cancer Statistics Public Use Research Data Agreement with the Centers for Disease Control and National Cancer Institute to access the de-identified cancer incidence data in the USCS database, and this study was exempt from ethical review.

**Figure 1.**
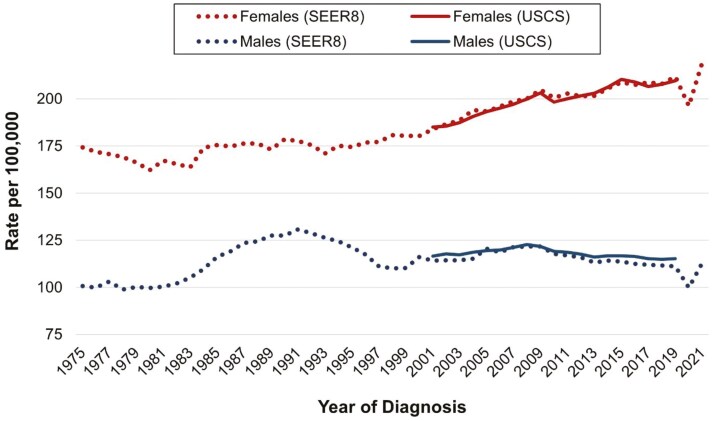
Overall early onset cancer incidence rates by sex, Surveillance Epidemiology and End Results 8 Registries (1975-2021) and United States Cancer Statistics Database (2001-2019). Abbreviation: SEER, Surveillance Epidemiology and End Results; USCS, United States Cancer Statistics. Annual incidence rates in adults, aged 20-49 years, are age-adjusted to the 2000 US standard population.

**Figure 2.**
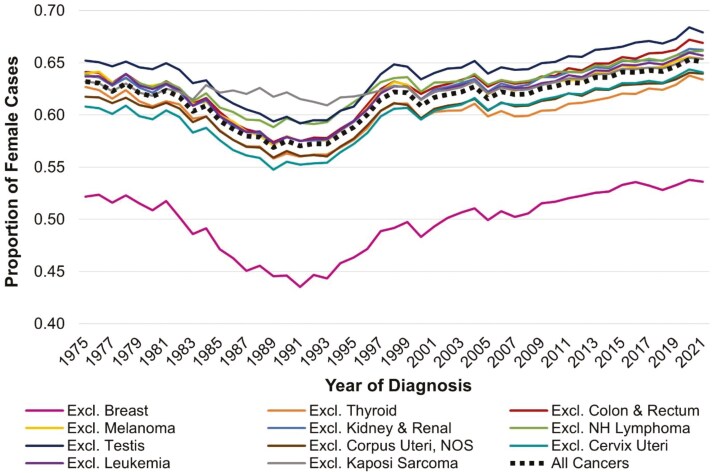
The proportion of incident early onset cancer cases that are in females, Surveillance Epidemiology and End Results 8 Registries, 1975-2021. Abbreviations: AAPC, average annual percent change; Excl., excluding; NH, non-Hodgkin lymphoma; NOS, not otherwise specified. The proportion of female cases was calculated by dividing the number of cancer cases in 20-49-year-old females by the total number of cancer cases in 20-49-year-old adults in each year from 1975 to 2021.

Figure 1 provides the overall incidence rates for early onset cancer in females and males and demonstrates similar trends between 2001 and 2019 in the SEER8 and USCS databases, supporting the use of SEER8 data to reflect national trends. The overall early onset cancer incidence rate in females increased from 117.5 per 100 000 in 2001 to 137.4 per 100 000 in 2019, representing a 17% increase in incidence. The overall early onset cancer incidence rate in males increased from 69.8 per 100 000 in 2001 to 74.5 per 100 000 in 2019, representing a 7% increase in incidence. **Figure 2** reveals that like the International Agency for Research on Cancer estimate for a single year, we also observed close to a 2/3 proportion of female cancers among cancers diagnosed in adults under 50 years for the majority of the last 45 years. The only period in which the proportion of female early-onset cancer cases dropped below 60% was between 1985 and 1995. This temporary decrease is likely attributed to the increase in acquired immunodeficiency syndrome (AIDS)-related cancers, such as Kaposi sarcoma.^13^ Since 1995, females have accounted for ≥60% of incident early-onset cancer cases, with the percentage increasing to 65% between 2019 and 2021. This recent increase in the proportion of female cases reflects the fact that females experienced an overall AAPC in early onset cancer incidence of 0.74% (95% CI = 0.62, 0.85) per year from 2001 to 2019. By contrast, there was no increase in overall cancers in males (−0.06% per year increase from 2001 to 2019;95% CI = −0.13, 0.02). Thus, while overall early-onset cancer incidence rates continued to increase in women in recent decades, rates remained stable on average in men.

When we examined the types of cancer that may be contributing to the sex difference in early-onset cancer incidence, we found that the difference was overwhelmingly attributed to the higher rates of breast cancer in women. We found that the proportion of female early-onset cancer cases dropped to around 50% (indicating parity between females and males) when we excluded breast cancer cases (the proportion ranged from 44% in 1991 and 1993 to 54% in 2016, 2020, and 2021). Three other cancers (uterine, cervical, and thyroid cancers) contributed to the higher proportion of females compared to male early onset cancer cases. However, excluding any one of these cancers only attenuated the female proportion by 1%-2%, supporting that breast cancer is the major driver of the sex difference in early onset cancer incidence.

**Figure 3.**
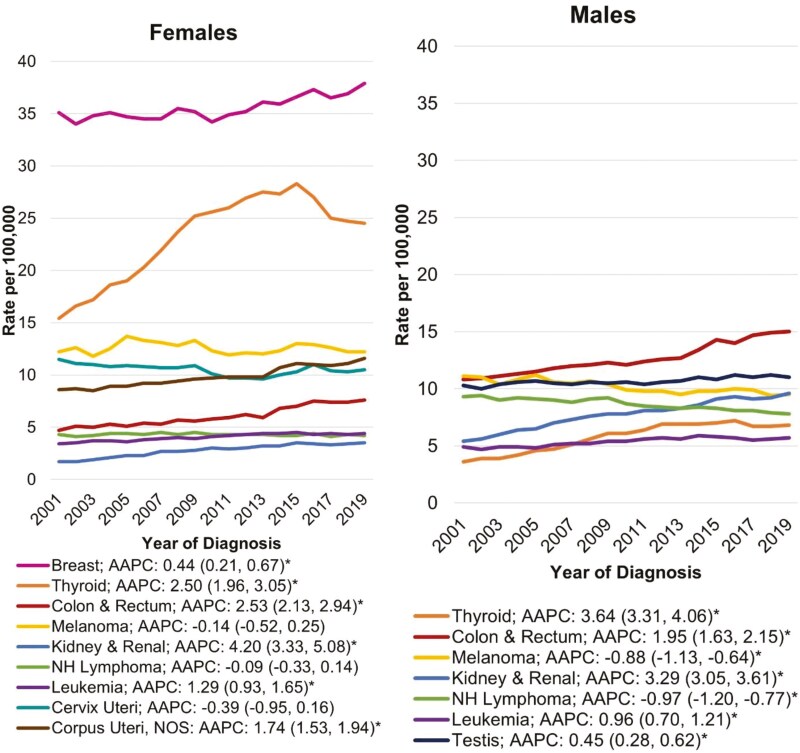
Early onset cancer incidence rates by sex and cancer type, United States Cancer Statistics Database, 2001-2019. Abbreviations: AAPC, average annual percent change; NH, non-Hodgkin lymphoma; NOS, not otherwise specified. Annual incidence rates in adults, aged 20-49 years, are age-adjusted to the 2000 US standard population. The AAPC for each cancer type was calculated using a weighted average of the slope coefficients of the underlying joinpoint regression line with weights equal to the length of each segment over the interval. AAPCs that were statistically different from zero (*P *< .05) are indicated with an asterisk.

As **Figure 3** shows, the top 2 highest incidence rates for females—breast and thyroid—are higher than any other single type of cancer in males. By contrast, incidence rates for colorectal cancer, kidney and renal pelvis cancer, leukemia, and non-Hodgkin lymphoma are higher in males than females under 50 years. Five of the top nine early-onset cancers in women increased in incidence from 2001 to 2019, including breast, thyroid, colorectal, kidney and renal pelvis, leukemia, and uterine cancers. Five of the top seven early-onset cancers in men increased from 2001 to 2019, including thyroid, colorectal, kidney and renal pelvis cancer, leukemia, and testicular cancer. Two of the top seven early-onset cancers in men decreased from 2001 to 2019, including melanoma and non-Hodgkin lymphoma. These findings support the importance of disaggregating cancer types when describing sex disparities in early-onset cancer incidence.

## Implications of sex-specific trends in early onset cancer incidence

It is not clear what is causing early-onset cancer incidence rates to change over time. Changes in screening practices cannot fully account for these trends, given that between 2001 and 2019 only breast and cervical cancers were screened for at a population level in women under age 50 years in the United States. The US Preventive Services Task Force recently updated their colorectal cancer guidelines to start screening at age 45 years,^14^ but this change is too recent to affect the trends over time. Furthermore, breast cancer screening does not begin until age 40 years in the United States, and we have shown a temporal increase in breast cancer incidence in US women between the ages of 25-39 years.^2^ The higher global rates in females under age 50 years were hypothesized to be partially because of the overdiagnosis of thyroid cancer in women due to their higher use of the medical system compared to men.^3^ Yet, at least in the United States, we showed that the higher rates of thyroid cancer in women under 50 years only account for 1%-2% of the sex difference in early onset cancer incidence. Therefore, other factors besides changes in screening and medical imaging technology are likely contributing to the increase in early-onset cancer.

In terms of risk factors that may be implicated in driving these early-onset cancers, just like with older onset cancers, they are likely to be multifactorial and differ by cancer type.^7^ For example, while the rise in childhood and adulthood obesity over time has likely contributed to the increase in gastrointestinal cancers in young adults,^2^ it cannot explain the increase in early onset breast cancer over time as childhood obesity is associated with lower premenopausal breast cancer risk.^15^ We have also shown that other established risk factors for breast cancer, such as age at first childbirth or parity cannot explain the increase in early onset breast cancer in the United States^16^ or globally^8^ Thus, there is a great need to develop evidence on the causes of these trends. Given the long latency of tumorigenesis, risk factors in early life, including environmental chemical exposures may play an important role in early-onset cancers of some cancers like breast.^17,18^ However, data are limited on whether early-life exposures contribute to the etiology of other early-onset cancers.

Addressing this research gap will likely require innovative analytic methods and study designs, given that it is difficult to study exposures across the life course in relation to rare diseases such as early-onset cancers. We have developed methods to look at time-series analyses of changes in risk factors with changes in cancer trends,^19^ which we have used to show that there are clustering of cancers based on organ systems.^20^ This approach of using a time-series ecological data for early onset cancers, which we have employed for other cancers like colorectal cancer,^5,21^ was recently endorsed by other cancer epidemiologists.^22^ Yet, while ecological studies are useful for hypothesis generation, other study designs are needed to examine individual risk factors. Cohort studies have traditionally been viewed as the study design of choice for etiological cancer studies. However, given the lower incidence of early onset cancers, enriched family studies^23^ or case–control studies^24^ may be a more efficient design for studying cancer risk in younger adults. For example, we previously conducted a nested case–control study in a cohort enriched for women with a family history of breast cancer to establish an association between exposure to polycyclic aromatic hydrocarbons and breast cancer risk using stored blood samples collected at cohort enrollment.^25^ Furthermore, now that many publicly available databases (eg, environmental exposure databases) can be used for exposure data through data linkage, case–control studies do not need to rely solely on self-report of exposures.^24^ Tissue collection may also be more feasible in case–control studies than cohorts, allowing for the measurement of somatic mutations and their interaction with or mediation of environmental exposures in cases.^24^ Given these advantages, case–control studies should not be overlooked as a key tool for studying the etiology of early-onset cancers.

The persistently high burden of cancer in young women has important clinical implications, as trends highlight the need for healthcare providers to educate themselves and their patients about the early signs and symptoms of cancer in younger adults. Furthermore, primary care providers and gynecologists, who typically serve as the first point of contact for women in the healthcare system, should evaluate younger patients for cancer risk factors, including environmental and lifestyle factors and family history of cancer. Importantly, the increase in early onset cancer means that healthcare providers should not just focus on individuals based on higher genetic risk, given their family history. They should also ensure that they have the necessary knowledge and training to discuss cancer risk with their younger patients, including options for genetic counseling, early screening, and risk reduction for those at high risk. Oncologists and other healthcare providers involved in breast cancer treatment should also be aware of and considerate toward the unique needs and preferences of younger patients, such as fertility preservation options when relevant.^26^ Increasing trends in early-onset cancers also highlight the possible need for a re-evaluation of current screening guidelines for younger adults. For example, in addition to beginning screening early in populations with a family history of cancer, it may be important to consider early screening in other high-risk populations such as those with high exposure to certain environmental carcinogens. The fact that the sex difference in early-onset cancer has remained relatively unchanged for over 4 decades underscores the urgent need to apply novel approaches in research, clinical practice, and public health policy to address the growing burden of early-onset cancers in the United States and globally.

In conclusion, early-onset cancer incidence is a growing public health issue that continues to disproportionately affect females. Over time, the incidence of several types of early-onset cancer has increased in both women and men. Yet, women have consistently accounted for approximately 2/3 of all cancers diagnosed in adults under 50. This sex difference translates into a staggering 62.9 per 100 000 excess cancer cases in females compared to males. This is particularly alarming, as a diagnosis of cancer at any time is life-altering but prior to midlife has tremendous implications on families, careers, and educational opportunities. Given that the sex difference is explained primarily by breast cancer, it is important to consider whether established risk factors, such as reproductive factors, can explain the increase. When we looked at the global incidence of breast cancer, we found that this increase remained even after adjusting for global fertility rates.^8^ The increase in early-onset cancer is also not likely to be explained by obesity trends as childhood and adolescent obesity does not increase premenopausal breast cancer risk.^27^ The stable sex ratio and increasing absolute rates of early onset cancers demand additional research into risk factors that have been understudied like environmental chemicals. While it is important to continue monitoring the various types of cancers that are increasing in both sexes, identifying and addressing risk factors contributing to the high rate of breast cancer in young women is critical for reducing the overall burden of early-onset cancer.
